# Dual targeted and pH-responsive gold nanorods with improved chemotherapy and photothermal ablation for synergistic cancer treatment

**DOI:** 10.1039/c8ra09422e

**Published:** 2019-02-14

**Authors:** Jing Wang, Hui Wang, Lin Yan, Zhiqiang Hu, Xiuli Wu, Fengmei Li

**Affiliations:** College of Pharmacy, Ningxia Medical University 1160 Shengli Street 750004 Yinchuan China jingwang_2018@163.com +86-951-408-1046 +86-951-408-1046; Key Laboratory of Hui Ethnic Medicine Modernization, Ministry of Education, Ningxia Medical University 659 Shengli Street 750004 Yinchuan China; Ningxia Hui Medicine Modern Engineering Research Center and Collaborative Innovation Center, Ningxia Medical University 659 Shengli Street 750004 Yinchuan China; Affiliated Hospital of Ningxia Medical University 804 Shengli Street 750004 Yinchuan China

## Abstract

Cancer is considered to be one of the leading causes of morbidity and mortality worldwide. A multifunctional nanosystem based on gold nanorods (GNRs) has demonstrated the potential to enhance therapeutic performance. In this research, dual-targeted pH-responsive GNRs for synergistic cancer treatment were developed and investigated. The GNRs could target angiogenic endothelial cells in the tumor region using αvβ3-mediated recognition and subsequently facilitate its specific binding to tumor cells mediated *via* recognition of the folate receptor, which could accumulate precisely at the tumor site. Doxorubicin (DOX) was loaded on to the surface of GNRs *via* a pH-sensitive hydrazone (hz) bond, which could effectively control the drug release by responding to the tumor acidic microenvironment. *In vitro*, the FA/RGD-DOX-*hz*-GNRs showed higher tumor specificity and killing ability under near-infrared irradiation. Furthermore, in B16-F10 xenograft tumor-bearing mice, FA/RGD-DOX-*hz*-GNRs produced the optimal tumor therapeutic efficacy by antagonizing angiogenesis, inhibiting cell proliferation and causing necrosis. Therefore, the strategy of integration of a photothermal effect, chemotherapy and a molecular active targeting based double-targeting mode appeared advantageous over chemotherapy or a photothermal therapy alone.

## Abstract 2

A dual-targeted pH-responsive GNR for synergistic cancer treatment was developed and investigated, which demonstrated the desired potential for enhancing therapeutic performance.

## Graphical abstract

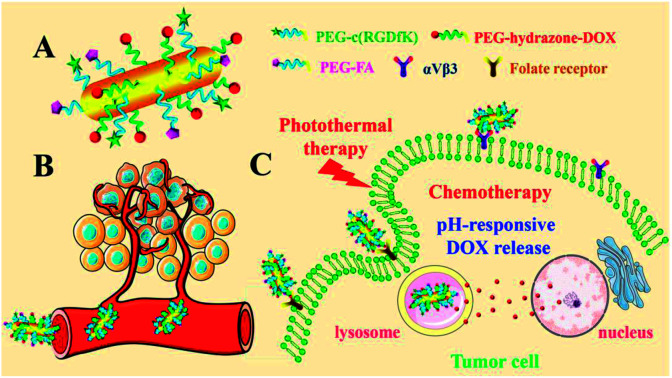

## 1. Introduction

Cancer has become one of the major threats to human health.^1^ Although current treatments such as surgical intervention, radiotherapy and chemotherapy can provide significant benefits, they can also cause collateral damage to surrounding tissues and adverse side-effects in patients.^2,3^ Development of highly integrated strategies for precise and efficient therapy of cancer has attracted much attention in the past decades.^4–7^ As a result of cancer heterogeneity, using a synergistic combination of chemotherapy and other therapies would be an optimized design for cancer treatment.^8–10^ Recently, studies have shown a synergistic antitumor effect when chemotherapy and thermotherapy are combined in nanomedicine.^11–14^ In particular, gold nanorods (GNRs) have shown great promise in cancer imaging, drug delivery and photothermal therapy (PTT), because of the advantages of shape-dependent physical/chemical properties, large-scale synthesis, excellent biocompatibility and easy functionalization.^15–20^ Furthermore, GNR mediated photothermal ablation therapy possesses minimal invasiveness and precise spatial-temporal selectivity in comparison with conventional therapeutic modalities, because its therapeutic effect happens only at the tumor site where both light absorbent and localized photo-irradiation coexist.^21,22^

Acid responsiveness, one of the most frequently used approaches among tumor environmental stimuli-triggered strategies in recent years,^23–25^ is the desired choice to achieve precise tumor targeting based on its intrinsic features in the tumor microenvironment and the existence of an evident pH gradient among blood vessels (pH 7.4), extracellular spaces (pH 6.5–7.2) and intracellular (pH 5.0–6.0) spaces in tumors.^26,27^ It is helpful to use a drug delivery nanovehicle to minimize non-specific systemic spread of toxic drugs while maximizing tumor directed drug delivery efficiency.^28^

However, the complicated physiological barriers in the tumor tissues and nonspecific interactions and recognition in normal tissues were still not overcome.^29,30^ Furthermore, aggregation is a common issue during preparation and application of a multifunctional nanosystem because of the interaction force between particles and a complicated modification of functionality, which may hamper precise transport of nanoparticles to the tumor site and limit its penetration into solid tumors.^31^

Therefore, an effective anticancer nanomedicine should not only display high cellular uptake, but also be able to exploit specific environmental stimuli to achieve effective release of the drug payload at target sites.^32–34^ More importantly, there is great demand to develop a nanosystem with more precise targeting of the tumor microenvironment for enhancing drug accumulation and improving the transport performance, and thus, the therapeutic efficacy.^35,36^

**Fig. 1:**
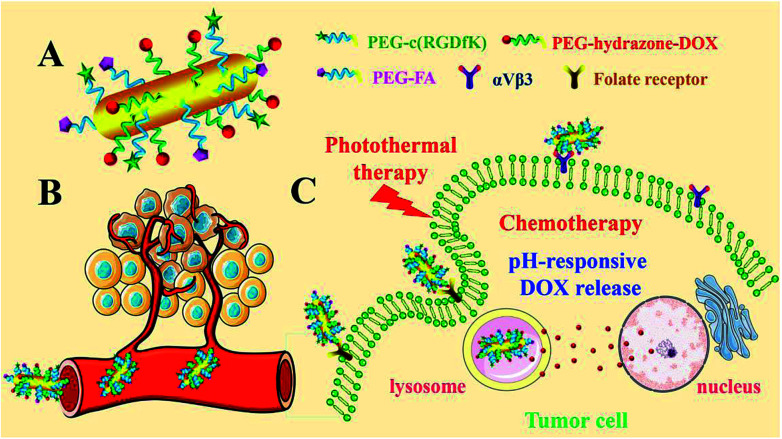
Illustration depicting FA/RGD-DOX-*hz*-GNR-associated targeted synergistic chemo-photothermal treatment of cancer.

In this paper, the development of dual targeted, pH-responsive nanomedicine built upon GNRs (Fig. 1) is reported. The cyclic arginine-glycine-aspartate (cRGD) and folic acid (FA) were decorated with poly(ethylene glycol) (PEG), with the aim of extending circulating time, reducing aggregation and escaping the reticuloendothelial system *in vivo*.^19,37,38^ Through recognition of vitronectin receptor (αvβ3), the nanorods could specifically target the angiogenic endothelial cells in tumor region, and subsequently, selectively bind to the tumor cells *via* recognition of the folate receptor (FR). Simultaneously, doxorubicin (DOX), a leading clinical anticancer agent, was conjugated to PEG passivated GNRs *via* a pH-sensitive hydrazone (hz) linkage to facilitate controlled release in the tumor microenvironment. The FA/RGD-DOX-*hz*-GNRs have the advantages of easy and effective large-scale synthesis, no aggregation in a complex physiological environment and highly targeted specificity to tumor tissues, which is favored for potential applications of targeted nanomedicine. Meanwhile, by integrating chemotherapy and PTT, the FA/RGD-DOX-*hz*-GNRs can synergistically improve and enhance antitumor therapeutic efficacy.

## 2. Experimental section

### 2.1. Materials

Chloroauric acid (HAuCl_4_·3H_2_O), cetyltrimethylammonium bromide (CTAB), sodium borohydride (NaBH_4_), silver nitrate (AgNO_3_), l-ascorbic acid (L-AA), hydrochloric acid (HCl), doxorubicin hydrochloride (DOX·HCl), 3-[4,5-dimethylthiazol-2-yl]-2,5-diphenyltetrazolium bromide (MTT), bisBenzimide H 33342 trihydrochloride (Hochest 33342), calcein acetoxymethyl ester (Calcein-AM) and propidium iodide (PI) were purchased from Sigma Co., Ltd. (USA). LysoTracker Green was purchased from Invitrogen Molecular Probes (USA). The c[RGDfK(maleimide)] peptide [Mal-c(RGD), MW = 736 Da] was purchased from Hefei Bank Peptide Ltd (China). The thiol-poly(ethylene glycol)-thiol (SH-PEG-SH, MW = 3400 Da), hydrazide-poly(ethylene glycol)-thiol (HY-PEG-SH, MW = 3400 Da), folate-poly(ethylene glycol)-thiol (FA-PEG-SH, MW = 3400 Da), rhodamine B-poly(ethylene glycol)-thiol (RB-PEG-SH, MW = 3400 Da) were purchased from Shanghai ToYong Bio. Tech. Inc. (China). Dimethyl sulfoxide and *N*,*N*-dimethylformamidee (DMF) were purchased from Shanghai Ling Feng Chemical Reagent Co., Ltd. (China).

The human umbilical vein endothelial cells (HUVEC) and mouse skin melanoma cell line (B16-F10) were obtained from purchased from the Shanghai Institute for Biological Sciences, Chinese Academy of Science. Dulbecco's Modified Eagle medium and fetal bovine serum were purchased from Gibco BRL (USA). Water was purified using distillation, deionization, and reverse osmosis (Millipore Milli-Q plus). All chemicals were analytical grade and were used as received without further purification.

### 2.2. Synthesis of GNRs

GNRs with a peak absorption wavelength of 822 nm were synthesized using a seed-mediated growth method.^18^ Briefly, a seed solution was prepared by mixing 7.5 mL of CTAB (0.1 M) and 2.5 mL of HAuCl_4_ (1 mM) with 0.6 mL of a freshly prepared NaHB_4_ (10 mM) solution with vigorous stirring at 28 °C. The seed solution was aged for 2 h before use. The growth solution was prepared by mixing 132 mL of 0.1 M CTAB with 120 mL of 1 mM HAuCl_4_ in a 100 mL flask. Afterwards, 2.4 mL of a 10 mM aqueous solution of AgNO_3_ and 2.2 mL of 2 M HCl were added to the contents of the flask. After gently mixing the solution, 1.92 mL of 0.1 M L-AA was added. Under continuous stirring, 0.5 mL of seed solution was finally added to initiate the growth of the GNRs. The GNRs were aged for 5 h to ensure full growth at 28 °C. The reaction products (CTAB-GNRs) were isolated using centrifugation at 10 000 rpm for 10 min followed by removal of the supernatant. The precipitates were re-dispersed in water.

### 2.3. Synthesis of DOX-hydrazone linked gold nanorods (DOX-*hz*-GNRs)

A portion of HY-PEG-SH (10 mM, 1 mL) was added into 9 mL of CTAB-GNR solution [optical density (OD) = 2], which was then stirred for 24 h. After centrifugation, the precipitates were dissolved in 10 mL of DMF, and an excess amount of DOX (10.8 mg), with reference to the drug binding hydrazide residues of PEG, was added. The mixed solution was stirred for 48 h with protected from light. The products (DOX-*hz*-GNRs) were obtained after repeated centrifugation.

### 2.4. Preparation of folate/c(RGDfK) decorated DOX-hydrazone linked gold nanorods (FA/RGD-DOX-*hz*-GNRs)

For further modification with targeted ligands, DOX-*hz*-GNRs were resuspended in 8 mL of phosphate buffer solution (PBS, pH = 7.4). To the solution, 2 mL of FA-PEG-SH and SH-PEG-SH mixed solution (5 mM, v : v = 1 : 1) was added and stirred under an argon atmosphere for 6 h. The GNRs were then collected using centrifugation and resuspended in PBS. After that, the maleimide conjugation reaction was carried out using Mal-c(RGD) (5 mM) with the thiol groups of the PEGylated GNRs. The final products (FA/RGD-DOX-*hz*-GNRs) were gathered and stored at 4 °C.

### 2.5. Stability assay and thermal responses of FA/RGD-DOX-*hz*-GNRs

After preparation, the FA/RGD-DOX-*hz*-GNRs were dispersed in cell culture medium. The short-term stability of FA/RGD-DOX-*hz*-GNRs was monitored at 3, 6, 9, 12 and 24 h. The long-term stability of the nanorods was monitored at 1, 2, 3, 5 and 7 d using ultraviolet-visible (UV-Vis) absorption spectroscopy.

To measure the temperature response of FA/RGD-DOX-*hz*-GNRs irradiated using a near-infrared (NIR) laser at 808 nm, 1 mL of FA/RGD-DOX-*hz*-GNRs aqueous suspensions (OD = 2) was placed in a single well of a 24-well plate and irradiated with a NIR laser from the top at different laser densities (2, 4 and 6 W cm^−2^). Meanwhile, the probe of a sensitive digital thermometer was immersed in the suspension, and the temperature response was recorded at intervals of 1 min.

### 2.6. The pH-responsive DOX release profile of FA/RGD-DOX-*hz*-GNRs

The pH-responsive DOX release from FA/RGD-DOX-*hz*-GNRs was evaluated in PBS with different pH values (pH = 5.0, 5.5, 6.0, 6.5, 7.0 and 7.4). In a typical experiment, 10 mL of FA/RGD-DOX-*hz*-GNRs suspension was loaded in a dialysis bag (MW 3500 cut off). The dialysis bag was immediately placed in 50 mL of a corresponding buffer at 37 °C. Periodically, 2 mL of the external buffer solution was taken out and replaced with an equal volume of fresh medium. The amount of DOX was quantified by measuring its absorbance at 488 nm against a standard curve. The absorbance was measured spectrophotometrically at 488 nm with a UV-Vis spectrophotometer to calculate the DOX release profile by comparison of the measured absorbance values against the total absorbance of free DOX.

### 2.7. Confocal laser scanning microscopy (CLSM) analysis of targeted cell uptake and intracellular DOX release

The binding, in particular that of FA/RGD-GNRs to integrin αvβ3 and the FR was determined using the cellular uptake in HUVEC and B16-F10, respectively. After HUVEC and B16-F10 cells were cultured in a monolayer in 35 mm confocal dishes, 100 μg mL^−1^ of RB-GNRs, RB-RGD-GNRs and RB-FA/RGD-GNRs were added and co-incubated with folate-free medium for different times (3, 6 and 9 h). The cells were washed three times with cold PBS and fixed with 4% paraformaldehyde in PBS for 15 min. The cells were then processed in a Hoechst 33342 stain for 20 min and then the amount of fluorescence determined using CLSM. The emission of RB was measured from 550 nm to 620 nm under the excitation of a 488 nm laser. All the images were obtained through a 60× oil objective.

To further locate the FA/RGD-DOX-*hz*-GNRs and evaluate the intracellular DOX release, B16-F10 cells were seeded into a 35 mm confocal dishes. After culturing overnight, 100 μg mL^−1^ of FA/RGD-DOX-*hz*-GNRs solution was co-incubated with the target cells for 3 h, then cells were gently washed twice with PBS, and incubated for another 3 h and 21 h in 2 mL of growth medium, followed by addition of LysoTracker Green and Hoechst 33342 according to the manufacturer's protocol. The CLSM analysis of these cells was performed, and all the images were obtained through a 100× oil objective.

### 2.8. Cytotoxicity assay and chemo-photothermal therapy *in vitro*

The cytotoxicity of the prepared FA/RGD-DOX-*hz*-GNRs was evaluated using the MTT assay. B16-F10 cells were routinely cultured before seeding into 96-well plates at a density of 1 × 10^4^ cells per well and incubated overnight. The cells were then treated with fresh medium containing free DOX, DOX-*hz*-GNRs (DOX = 1 μg mL^−1^) and FA/RGD-DOX-*hz*-GNRs (DOX = 2 μg mL^−1^). After 24, 48 and 72 h of incubation, the absorbance was monitored using a microplate reader at a wavelength of 490 nm with a reference wavelength of 630 nm as designated in the protocol of the MTT assay. The cell viability was obtained by normalizing the absorbance of the sample against that from the control well and expressing it as a percentage, assigning the viability of non-treated cells as 100%.

The photothermal and chemo-photothermal effect were also evaluated, and B16-F10 cells were incubated with 100 μg mL^−1^ of FA/RGD-GNRs and FA/RGD-DOX-*hz*-GNRs for about 4 h before laser irradiation. Then the cells were washed twice with PBS and replenished with fresh complete medium. Afterwards, the cells were irradiated at 808 nm with a 5 mm diameter spot size at 4 W cm^−2^ for 5, 10 and 15 min. Next, the cells were incubated for another 12 and 24 h at 37 °C. The cell viability was measured using the MTT assay. Meanwhile, another portion of the treated cells was incubated with Calcein-AM and PI for 20 min at 37 °C according to the protocol. The cell images were captured using a fluorescence microscope.

### 2.9. *In vivo* studies: antitumor efficacy determination

Male C57BL/6 mice (4–6 weeks old, 18–22 g) were purchased from Beijing Weitong Lihua Experimental Animal Technology and received care in the Laboratory of the Animal Center, Ningxia Medical University. All animal procedures were performed in accordance with the ‘*Guidelines for Care and Use of Laboratory Animals of Ningxia Medical University*’ and the experiments were approved by the Animal Ethics Committee of Ningxia Medical University. To generate the melanoma xenograft tumor model, B16-F10 cells (1 × 10^6^) were injected subcutaneously into the right rear flank area of each mouse. After 7–10 d, mice with tumors exceeding 60 mm^3^ in volume were randomly divided into five groups (*n* = 10). Normal saline (NS; 200 μL), DOX (2.5 mg kg^−1^), FA/RGD-GNRs and FA/RGD-DOX-*hz*-GNRs (containing 2.5 mg kg^−1^ of DOX) were intravenously administered to the mice after 2, 6 and 9 d. The NS, FA/RGD-GNRs and FA/RGD-DOX-*hz*-GNRs groups were exposed to 4 W cm^−2^ NIR light for 15 min 12 h post-injection. The tumor volume and body weight of each mouse were monitored every 3 d for 18 d. Tumor volumes were measured using a digital caliper and calculated using the following formula: *V* = *L* × *W*^2^/2 (*W*: the shortest dimension; and *L*: the longest dimension). At the end of the experiment, the mice were sacrificed and tumors from different groups were collected and fixed with 10% neutral buffered formalin and embedded in paraffin. The tumor histological slices were stained with hematoxylin and eosin (H&E). Also, after removal of melanin, the embedded tissues were used for immunohistochemistry analyses of Ki-67 and CD 34 protein expression. The tumor cross-sections were observed with a light microscope.

### 2.10. Statistical analysis

All the experiments were performed in triplicate (minimum number) and expressed as means ± standard deviation. The differences among the groups were determined using the paired, two-sided Student's *t*-test. A *P*-value less than 0.05 was considered to be significant, and a *P*-value less than 0.01 was considered to be highly significant.

## 3. Results and discussion

### 3.1. Preparation and characterization of FA/RGD-DOX-*hz*-GNRs

**Fig. 2:**
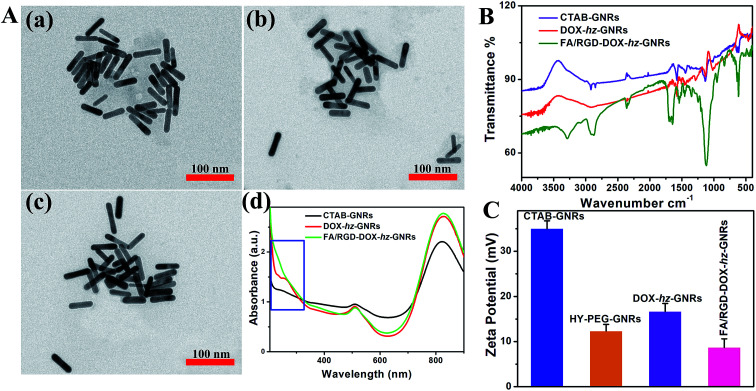
Characterization of GNRs. (A) TEM images [(a)–(c)] and UV-Vis-NIR spectra (d) of CTAB-GNRs, DOX-*hz*-GNRs and FA/RGD-DOX-*hz*-GNRs, respectively. (B) FTIR spectra of CTAB-GNRs, DOX-*hz*-GNRs and FA/RGD-DOX-*hz*-GNR. (C) Zeta potentials of CTAB-GNRs, HY-PEG-GNRs, DOX-*hz*-GNRs and FA/RGD-DOX-*hz*-GNRs.

The design and preparation of FA/RGD-DOX-*hz*-GN*Rs* is illustrated in Fig. 1. The morphology of the GNRs was characterized using transmission electron microscopy (TEM) (Fig. 2A), and the TEM images revealed that the average length and width of the bare GNRs was 45 ± 2.5 nm and 12 ± 1.2 nm, respectively, (approximately 3.75 : 1 aspect ratio) (Fig. 2A(a)). The FA/RGD-DOX-*hz*-GNRs nanoplatforms were finally fabricated using surface functionalization of GNRs with DOX, folate and c(RGD) on the heterobifunctional PEGs. The conjugation of functional molecules had little impact on the dimensions of GNRs (Fig. 2A(b and c)), suggesting that the nanorods were not affected by the applied conjugation chemistry. As shown in Fig. 2A(d), the absorption spectra of the GNRs were investigated, all of which possessed transverse peaks at 510 nm. After DOX conjugation mediated using hz-linked chemistry, the longitudinal peaks at 822 nm experienced a slight red-shift to 828 nm. Meanwhile, the absorbance of DOX at 233 nm and 253 nm was also observed, with an obvious increase at 828 nm suggesting the change of near-distance dielectric sensitivity around the GNRs. Similarly, the conjugation of targeted ligands further increased the longitudinal absorbance of GNRs and changed the waveform of the FA/RGD-DOX-*hz*-GNRs between 200–300 nm.

The DOX loading and targeted ligands' conjugation were also determined using Fourier-transform infrared (FTIR) measurements (Fig. 2B). The presence of obvious O–H bands (∼3450 cm^−1^) and C=C bands (∼1617 cm^−1^) demonstrated that DOX was successfully loaded on to the surface of CTAB-GNRs using hz-linked chemistry. Furthermore, the strong stretching vibration of N–H–C=O (∼1658 cm^−1^), –COOH (∼1693 cm^−1^) and N–H bands (∼3290 cm^−1^and 1640 cm^−1^) indicated that folate and c(RGD) modification was available through the heterobifunctional PEGs.

The surface charge of the nanorods after the modification was monitored by measuring the zeta potential (Fig. 2B). The zeta potential for the CTAB-GNRs was 35 ± 1.7 mV, because of the presence of an amount of CTAB molecules. Through the robust Au-S bond formation, the surface charge of HY-PEG-GNRs was reduced to 12 ± 1.4 mV. Although the amines in the hydrazide group of heterobifunctional PEGs were positively charged, ligand replacement of CTAB was more helpful in decreasing the surface charge. With the addition of the antitumor agent, DOX, the charge slightly increased to 16 ± 1.7 mV. The net positive charge on the final FA/RGD-DOX-*hz*-GNRs after targeted modification was 8 ± 1.9 mV, which was attributed to the carboxylation of folate and c(RGD).

### 3.2. Stability, thermal response and DOX release behavior of FA/RGD-DOX-*hz*-GNRs

**Fig. 3:**
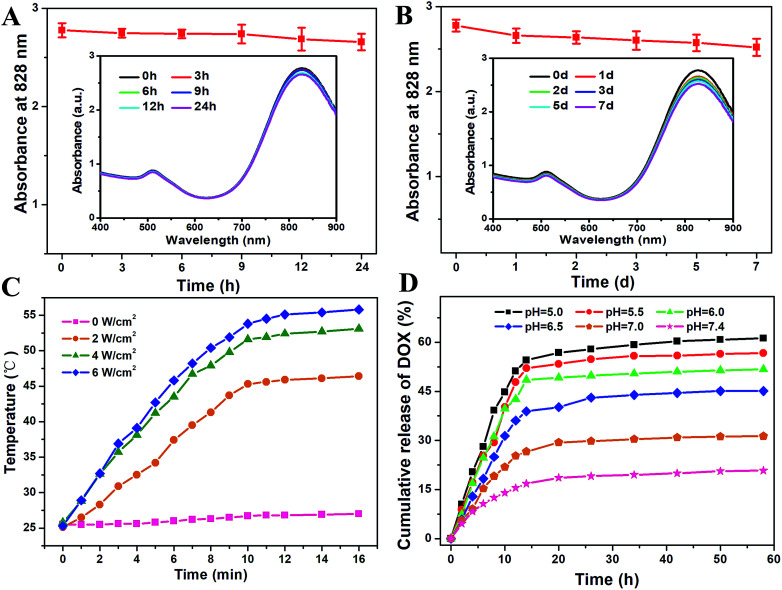
The stability, temperature response and DOX release of FA/RGD-DOX-*hz*-GNRs. (A and B) The short-term and long-term stability of FA/RGD-DOX-*hz*-GNRs in culture medium. (C) The temperature response of FA/RGD-DOX-*hz*-GNRs with varying NIR laser power density. (D) DOX release from FA/RGD-DOX-*hz*-GNRs in buffers at different pH values.

The functional GNRs tend to aggregate in a complex physiological environment. Thus, the stability of FA/RGD-DOX-*hz*-GNRs should be carefully considered before using them in *in vitro* and *in vivo* applications. As seen in Fig. 3, the heterobifunctional PEG modification retained the stability of GNRs. More importantly, the featured longitudinal peak at 828 nm in physiological medium was maintained and decreased slightly with incubation for 24 h (Fig. 3A). Also, it should be noted that the desired biocompatibility of the nanorods was maintained in a biological environment for up to 7 d (Fig. 3B), indicating no aggregation or destruction of GNRs.

In order to examine the GNRs with a high thermal radiation response, FA/RGD-DOX-*hz*-GNRs were exposed to NIR laser radiation with an increased power density. Fig. 3C shows that the solution temperature increased rapidly with the increasing NIR radiation power density and the extended irradiation time, confirming that GNR complexes could convert NIR light energy into heat efficiently and quickly. More significantly, after irradiation for 10 min, the solution temperature exceeded 45.3 °C with a relatively low power density of 2 W cm^−2^, and 51.6 °C with a feasible power density of 4 W cm^−2^, which was favourable for inducing apoptosis, and even necrosis, of tumor cells treated with GNRs upon PTT.^15,39,40^

To evaluate the pH sensitivity of FA/RGD-DOX*-hz*-GNRs *in vitro*, a DOX release experiment was performed at 37 °C in PBS at pHs of 5.0, 5.5, 6.0, 6.5, 7.0 and 7.4. As shown in Fig. 3D, the release rates of DOX from the GNRs were remarkably affected by the pH values of the microenvironment. Compared with 61.2% obtained at pH 5.0 after 58 h release, the cumulative release of DOX from FA/RGD-DOX-*hz*-GNRs at pH 7.4 was estimated to be 20.8%, and no obvious burst release was observed indicating comparative stability in neutral conditions. The DOX release was accelerated upon decreasing the pH values to 6.5, 6.0 and 5.5, and the cumulative release was 45.1%, 51.7%, and 56.7%, respectively, implying the breakage of the hydrazone bond occurred in acidic conditions. These results demonstrated that the hydrazone-linked GNRs when internalized by tumor cells were able to efficiently release DOX in the endo/lysosomal compartments and that they were stable at a physiological environment. Therefore, it is thought that the FA/RGD-DOX-*hz*-GNRs had a pH stimulus drug release property, which was desirable for targeted cancer therapy and provided a foundation for its application *in vivo* within an acidic tumor environment.

### 3.3. Targeting intracellular uptake and distribution

**Fig. 4:**
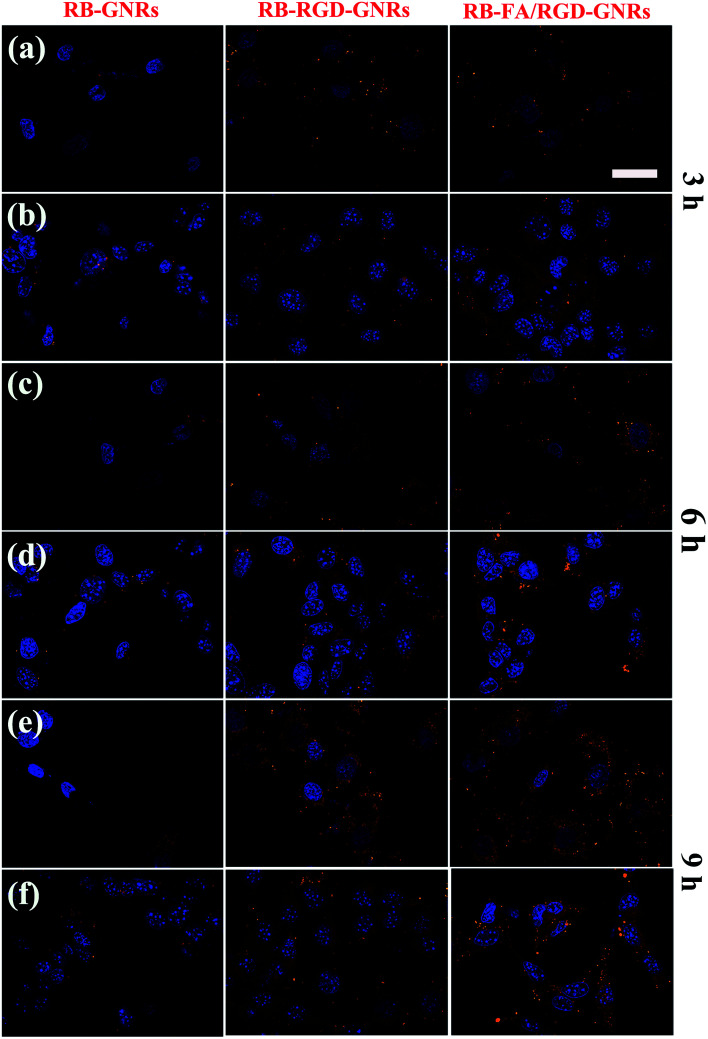
Targeting cell uptake of RGD-GNRs and FA/RGD-GNRs. (a, c and e) CLSM images of HUVEC cells incubated with RB-GNRs, RB-RGD-GNRs and RB-FA/RGD-GNRs for 3, 6 and 9 h. (b, d and f) CLSM images of B16-F10 cells incubated with RB-GNRs, RB-RGD-GNRs and RB-FA/RGD-GNRs for 3, 6 and 9 h. (Scale bar = 50 μm).

To investigate the cellular uptake of targeted GNRs with single ligand and dual ligands more intuitively and effectively, the fluorescence intensity of RB was observed using CLSM with unmodified GNRs as the control (Fig. 4). After incubation with RB-GNRs, RB-RGD-GNRs and RB-FA/RGD-GNRs for 3 h, varying amounts of nanorods were taken up by cellular internalization. In the cytoplasm of HUVEC, the fluorescence intensity of RB-RGD-GNRs and RB-FA/RGD-GNRs was similar, indicating the interaction between the single ligand mediated targeting through recognition of αvβ3, overexpressed in angiogenic endothelial cells.^6^ Furthermore, the fluorescence intensity of the RB-FA/RGD-GNRs was markedly higher than that of the RB-RGD-GNRs and RB-GNRs in B16-F10 cells, demonstrating the effectiveness of dual ligand mediated targeting because their corresponding receptors, αvβ3 integrin and FR, were overexpressed in malignant tumor cells.^12^ By extending the incubation time to 9 h, more functional nanoparticles were delivered into the cytoplasm of targeted cells by receptor mediated endocytosis. A stronger fluorescence signal of RB-FA/RGD-GNRs was captured in B16-F10 cells than that in HUVEC cells, suggesting that further modification with FA could improve cellular internalization of the nanorods. Therefore, the fabricated dual ligand targeted GNRs could selectively and sequentially target angiogenic endothelial cells and malignant tumor cells with αvβ3 integrin and FR overexpression in tumor tissues.

**Fig. 5:**
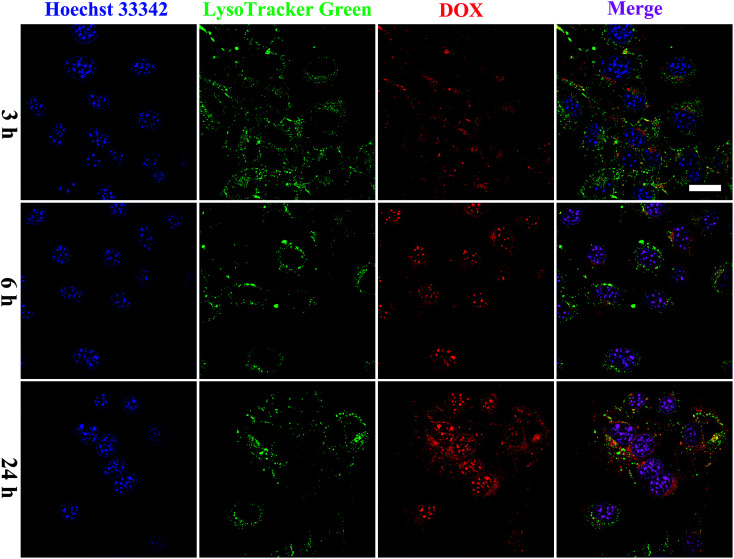
Intracellular distribution of FA/RGD-DOX-*hz*-GNRs. CLSM images of B16-F10 cells incubated with FA/RGD-DOX-*hz*-GNRs for 3, 6 and 24 h. The lysosome was labeled with LysoTracker Green and the nuclei were stained with Hoechst 33342. (Scale bar = 20 μm).

To further examine the intracellular distribution of FA/RGD-DOX-*hz*-GNRs and acid-triggered DOX release in B16-F10 cells, the fluorescence characteristics of DOX were used to monitor its intracellular distribution. The lysosomes and nucleus of the cells were stained with LysoTracker green and Hoechst 33342 immediately before the CLSM measurements (Fig. 5). After 3 h of co-culture, a weak fluorescence signal of DOX derived from FA/RGD-DOX-*hz*-GNRs was detected because of the limited DOX release in such a short time. Some studies showed that the fluorescence of DOX was markedly quenched once it was conjugated to the GNRs and as a result of the nanosurface energy transfer effect, the recovery of fluorescence observed inside the cells indicated intracellular DOX release.^41^ With the incubation time increased to 6 h, most of red fluorescence dots were located in the cytoplasm and overlapped with the green fluorescence of LysoTracker, suggesting that the FA/RGD-DOX-*hz*-GNRs were localized in the lysosomes and that DOX was released gradually inside the lysosomes. A slight red fluorescence was also observed in the nuclei, indicating that released DOX was transported into the cell nucleus. These results were consistent with those of the DOX release at pH 5.0 in the *in vitro* study. When the incubation time reached 24 h, a clearer fluorescence of DOX was observed and overlapped nearly all the green fluorescence stained regions. Furthermore, a higher intensity of red fluorescence DOX was detected in the cell nucleus and used as a DNA intercalator in the nuclei. The previous data suggest that FA/RGD-DOX-*hz*-GNRs were increasingly pushed into lysosomes and released the DOX in the lysosomes into the nucleus with time.

According to the findings described previously, the FA/RGD-DOX-*hz*-GNRs could effectively release DOX in to the microenvironment of tumor cells and which reduced undesired residence during circulation, which might greatly enhance the efficacy of cancer treatment.

### 3.4. *In vitro* cytotoxicity and chemo-photothermal therapy

**Fig. 6:**
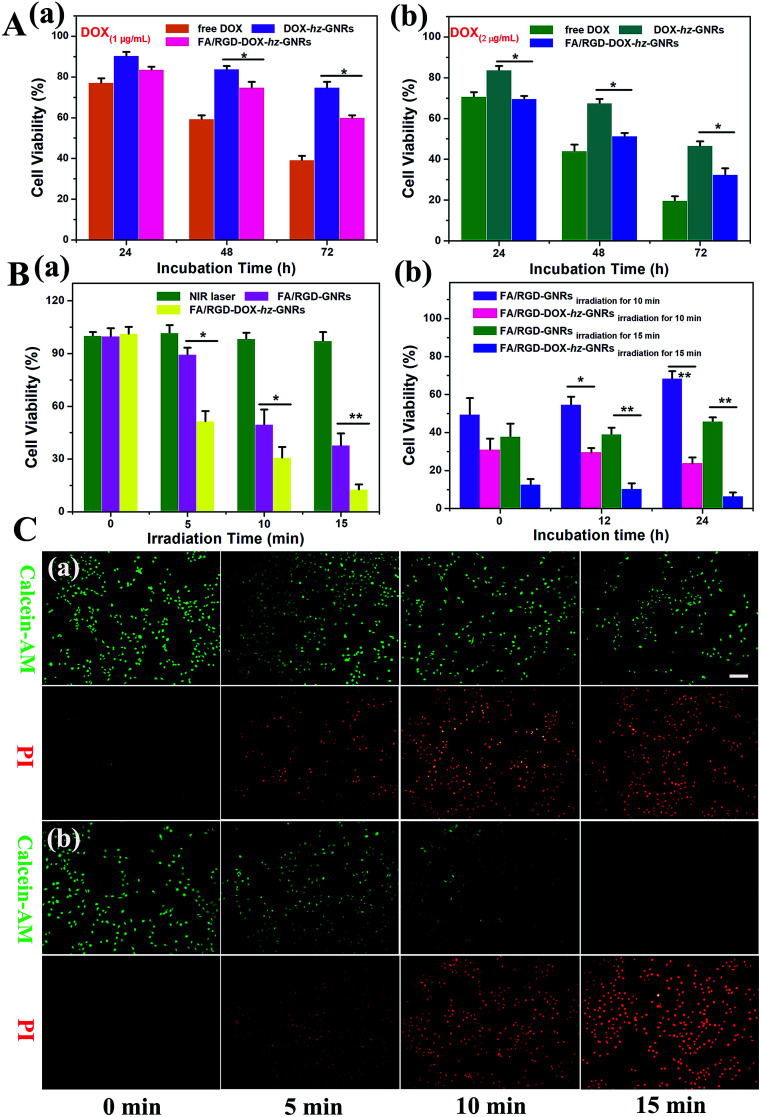
*In vitro* cell viability of monotherapy and combined therapy. (A) Viability of B16-F10 cells after incubation with free DOX, DOX-*hz*-GNRs and FA/RGD-DOX-*hz*-GNRs for 24, 48 and 72 h at an equivalent DOX concentration of 1 μg mL^−1^ (a) and 2 μg mL^−1^ (b). (B) Viability of B16-F10 cells after incubation with FA/RGD-GNRs and FA/RGD-DOX-*hz*-GNRs for 4 h with exposure to an 808 nm laser light for 5, 10 and 15 min (a) or cells irradiated for 10 and 15 min at different maintenance durations (b). (C) Thermal therapy and chemo-thermal combined treatment of tumor cells. Representative images of B16-F10 cells after incubation with FA/RGD-GNRs (a) and FA/RGD-DOX-*hz*-GNRs (b) for 4 h and exposed to a NIR laser. Live cells were stained with Calcein-AM and dead cells were stained with PI. (*P < 0.05, **P < 0.01, scale bar = 100 μm).

The cytotoxicity of free DOX, DOX-*hz*-GNRs and FA/RGD-DOX-*hz*-GNRs towards B16-F10 cells was evaluated using a standard MTT assay. As shown in Fig. 6A(a), compared with an inhibitory rate of about 9.7 ± 1.9% observed with DOX-*hz*-GNRs treatment, the FA/RGD-DOX-*hz*-GNRs reduced cell viability by 16.5 ± 1.5% after incubation for 24 h, implying that there was a negligible effect on cell viability. With the increases in the concentration of DOX and the incubation time, FA/RGD-DOX-*hz*-GNRs significantly increased the inhibition rate to 67.6 ± 3.1% (*P* < 0.05) compared with a 53.4 ± 2.2% reduction of DOX-*hz*-GNRs after 72 h incubation at an equivalent DOX concentration of 2 μg mL^−1^ (Fig. 6A(b)). The increasing cytotoxicity of FA/RGD-DOX-*hz*-GNRs was attributed to the dual ligand specific targeting to tumor cells. It is worth noting, however, that the antitumor activity of free DOX was highest and reached up to 80.4 ± 2.2%, which can be explained by the fact that FA/RGD-DOX-*hz*-GNRs entered the B16-F10 cells by receptor-mediated endocytosis, followed by the controlled release of DOX from the acid labile linker in the endosomal compartments, whereas free DOX could rapidly transfer into cells by passive diffusion. These results indicated that the conjugation of DOX with the PEGylated GNRs *via* an acid sensitive hydrazone bond could maintain the antitumor activity of the DOX well.

Next, the photothermal and chemo-photothermal therapeutic effect of FA/RGD-GNRs and FA/RGD-DOX-*hz*-GNRs towards B16-F10 cells induced by NIR laser were assessed *in vitro*. Fig. 6B(a) shows that no influence on cell viability of the control group was found, indicating that the photocytotoxicity was not obvious because of the minimal temperature elevation of the cell culture medium upon NIR irradiation. Cells treated with FA/RGD-GNRs and FA/RGD-DOX-*hz*-GNRs under 808 nm radiation exhibited time-dependent anti-tumor activity. The cell viabilities of FA/RGD-GNRs after laser irradiation for 5, 10 and 15 min were 89.4 ± 3.9%, 49.5 ± 8.4% and 37.8 ± 6.8%, which were higher than those of FA/RGD-DOX-*hz*-GNRs with 51.5 ± 5.8% (*P* < 0.05), 30.8 ± 6.0% (*P* < 0.05) and 12.6 ± 2.9% (*P* < 0.01), respectively. The increase of inhibition rate with extended irradiation time indicated accumulated phototoxicity of GNRs upon continued laser irradiation because of the elevated temperature, and this was in good agreement with the results of temperature response shown in Fig. 3C. However, B16-F10 cells administrated with FA/RGD-DOX-*hz*-GNRs produced a significant increase of inhibition rate compared with that obtained with FA/RGD-GNRs, which was attributed to synergistic chemo-thermal therapeutic effects on the cancer cells. Also, the cells irradiated for 10 and 15 min were then maintained for different periods of time. As shown in Fig. 6B(b), 24 h post-laser irradiation, the viabilities of the FA/RGD-GNRs-treated cells with exposure to a NIR laser for 10 and 15 min were 68.4 ± 3.9% and 45.9 ± 2.1%, indicating that there was incomplete eradication of cells by PTT alone. In contrast, the viability of FA/RGD-DOX-*hz*-GNRs-treated cells gradually decreased from 30.8 ± 6.0% to 23.7 ± 3.2% (*P* < 0.01) and 12.6 ± 2.9% to 6.4 ± 2.0% (*P* < 0.01), for 10 and 15 min irradiation, respectively, suggesting that the synchronous combinational therapy possessed a stronger killing cell ability than chemotherapy and PTT alone. The laser irradiation induced photothermal cytotoxicity was further visualized using a live-dead double staining assay (Fig. 6C). Calcein-AM used for the staining assay can only be activated in live cells to produce strong green fluorescence. The PI, a red fluorescence dye, was only able to penetrate dead cells because of the loss of membrane integrity. All the fluorescence images confirmed a higher cytotoxicity of FA/RGD-DOX-*hz*-GNRs than that of FA/RGD-GNRs after 5, 10 and 15 min of irradiation treatment, implying synergistic toxicity between DOX and a laser irradiation induced hyperthermia effect. These findings indicated that FA/RGD-DOX-*hz*-GNRs could either control the release of DOX in a tumor acidic microenvironment or efficiently convert NIR light into heat, thus, reducing the viability of tumor cells *in vitro*.

### 3.5. Synergistic antitumor effect with a combination of chemotherapy and photothermal therapy *in vivo*

**Fig. 7:**
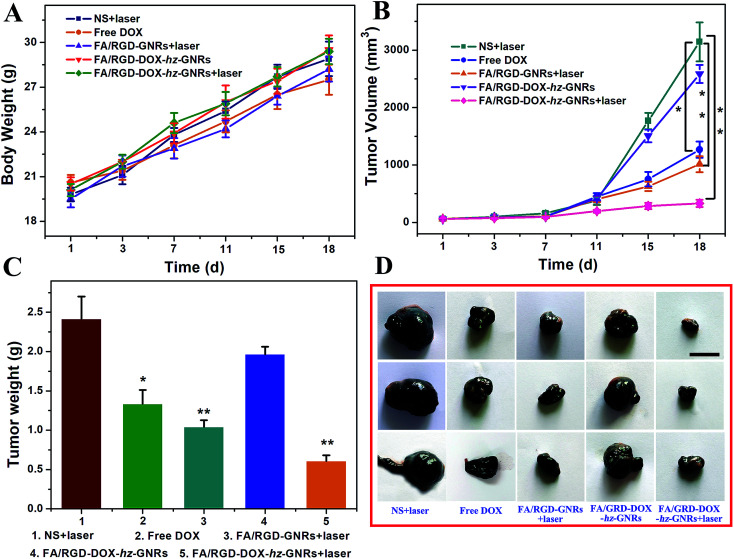
*In vivo* chemo-thermal combined tumor therapy with FA/RGD-DOX-*hz*-GNRs. (A) Body weight growth curves for mice treated with normal saline plus NIR laser, free DOX, FA/RGD-GNRs plus NIR laser, FA/RGD-DOX-*hz*-GNRs and FA/RGD-DOX-*hz*-GNRs plus NIR laser. (B) Changes in tumor volume *versus* time after different treatments. (C) Tumor weights of mice after treatment. (D) Representative photographs of tumors taken at day 18. (*P < 0.05, **P < 0.01, scale bar = 1 cm, *n* = 6 tumors).

The therapeutic efficacy of FA/RGD-DOX-*hz*-GNRs was evaluated in mice with a subcutaneously inoculated B16-F10 tumor. As shown in Fig. 7, during the treatment, no obvious change in the weight of the mice was observed from any of the treated groups suggesting that the experimental treatments at the test dose were well tolerated (Fig. 7A). The growth of tumors was monitored by measuring the tumor volume during the therapeutic period (Fig. 7B). Mice treated with NS plus laser and FA/RGD-DOX-*hz*-GNRs had a similar and rapid tumor growth, indicating that laser irradiation only or FA/RGD-DOX-*hz*-GNRs could not effectively inhibit tumor development. A significant reduction of tumor volume was observed for the mice treated with FA/RGD-GNRs plus laser irradiation (*P* < 0.01), confirming that targeted modified GNRs retained NIR light thermal conversion properties well *in vivo*. It is worth noting that DOX could not effectively inhibit tumor growth because the dose given was lower than the clinical treatment dose, thus, there was no significant systemic toxicity found in this experiment. The best tumor growth inhibition profile was found in the combination of FA/RGD-DOX-*hz*-GNRs and 808 nm NIR irradiation (Fig. 7B–D) (*P* < 0.01), demonstrating a synergistic tumor growth inhibition effect between laser irradiation and DOX treatment and simultaneously suggesting the dominant contribution of a laser irradiation-induced hyperthermia effect.

**Fig. 8:**
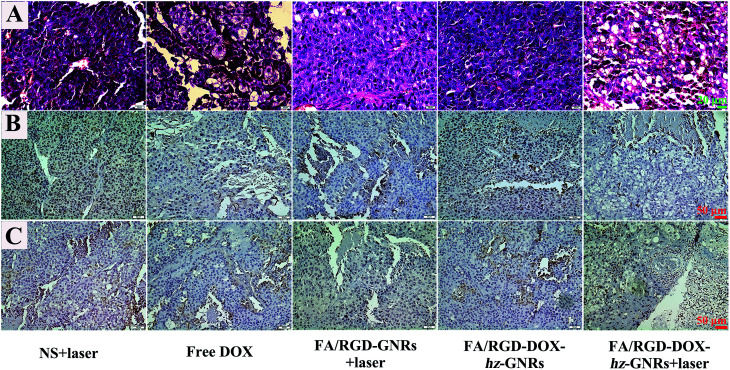
H&E staining and immumohistochemical staining of tumor tissues after treatment. (A) H&E staining of tumor tissues. (B) and (C) The expression of Ki-67 and CD 34 in tumor tissues, respectively.

The H&E analysis of the tumor further confirmed that after treatment with FA/RGD-DOX-*hz*-GNRs plus NIR laser irradiation, the tumor tissues exhibited the highest level of necrosis and the tumor cells were irregularly shaped with severely damaged cell membrane and shrinking nucleus (Fig. 8A). However, the cells retained their regular cell morphology with an intact nucleus in the NS plus laser groups. Furthermore, the cells in the group receiving FA/RGD-GNRs injection and NIR laser irradiation showed more obvious necrosis with significant vacuolation and chromatin condensation in contrast to the FA/RGD-DOX-*hz*-GNRs treated tumor, suggesting that the laser irradiation-induced hyperthermia effect was the major reason for the cellular necrosis. In addition, the Ki-67 and CD 34 immunohistochemistry staining assay was performed to determine the proliferation activity of the tumor cells and the microvessel density (MVD) in the sections of tumors. As shown in Fig. 8B, most tumor cells possessed high proliferation activity with Ki-67 positively staining in the NS plus laser group. However, the FA/RGD-DOX-*hz*-GNRs-guided combined therapy demonstrated the lowest cellular proliferation activity compared with cells treated with free DOX or FA/RGD-GNRs plus laser, indicating the synergistic therapeutic effect of chemo-thermal therapy compared to that with either chemotherapy or PTT alone. Analogously, the MVDs were significantly reduced after laser irradiation in either the FA/RGD-DOX-*hz*-GNRs or FA/RGD-GNRs treated groups (Fig. 8C). Nevertheless, only a slight MVD decrease was observed in DOX and FA/RGD-DOX-*hz*-GNRs without laser groups, which was in good agreement with the results of the Ki-67 assay. Angiogenesis plays an important role in the growth and metastasis of a tumor. Therefore, anti-angiogenesis is of great significance in tumor treatment. In the current study, FA/RGD-DOX-*hz*-GNRs could specifically target the angiogenic endothelial cells in the tumor region through recognition of αvβ3, which inhibited angiogenesis to initially block the tumor progression.

Collectively, these *in vivo* results showed that the validity of integration of photothermal effect, chemotherapy and molecular active targeting based double-targeting strategy, and combination therapy enabled by using FA/RGD-DOX-*hz*-GNRs possessed the best therapeutic effect *via* the angiogenesis inhibition ability and the tumor growth inhibition ability, both separately and simultaneously.

## 4. Conclusions

In summary, a dual-targeted pH-responsive nanomedicine was successfully fabricated which was built upon GNRs displaying excellent biocompatibility, tumor targeting specificity, controlled drug release ability and photothermal effect. Cellular uptake and location studies indicated that FA/RGD-DOX-*hz*-GNRs had a relatively high uptake against B16-F10 cells *via* integrin receptor and folate receptor mediated endocytosis, and more importantly, cellular internalized nanorods were sub-localized partly in the lysosomes, where DOX could be effectively released and which reduced undesired residence during circulation. The *in vitro* and *in vivo* antitumor activity studies confirmed the advantageous therapeutic efficacy of combined chemo-photothermal therapy upon FA/RGD-DOX-*hz*-GNRs, which could be attributed to a synergistic effect with the DOX-induced apoptosis, NIR laser irradiation-caused necrosis and angiogenesis inhibition. Thus, the results convincingly demonstrated that the dual-targeted pH-responsive drug delivery system based on GNRs has great potential in targeting and synergistic chemo-photothermal cancer therapy.

## Conflicts of interest

The authors declare no competing financial interest.

## Supplementary Material
